# Psychosocial work characteristics and sleep quality among early career registered nurses: a cross-sectional latent profile analysis

**DOI:** 10.1186/s12913-023-09949-9

**Published:** 2023-09-21

**Authors:** Katri Lönnqvist, Timo Sinervo, Anu-Marja Kaihlanen, Katri Vehviläinen-Julkunen, Marko Elovainio

**Affiliations:** 1 Doctoral Programme in Population Health, Faculty of Medicine, University of Helsinki, P.O. Box 63, Helsinki, 00014 Finland; 2 Finnish Institute for Health and Welfare, P.O. Box 30, Helsinki, 00271 Finland; 3 Department of Nursing Science, Faculty of Health Sciences, University of Eastern Finland, P.O. Box 1627, Kuopio, 70211 Finland; 4 Kuopio University Hospital, Puijonlaaksontie 2, Kuopio, 70200 Finland; 5 Department of Psychology and Logopedics, Faculty of Medicine, University of Helsinki, P.O. Box 21, Helsinki, 00014 Finland

**Keywords:** Organizational justice, Job demand, Job control, Sleep quality, Early career registered nurse, Latent profile analysis

## Abstract

**Background:**

Individual psychosocial work characteristics have been associated with health and well-being of registered nurses. However, it is yet to be determined whether different types of psychosocial work characteristics form patterned profiles and how these profiles are associated with the health and well-being. The purpose of this study was to identify latent psychosocial work characteristic profiles, including procedural, interactional and distributive justice, job demand and job control, and examine whether the profiles are associated with sleep quality among early career registered nurses.

**Methods:**

We conducted a cross-sectional study comprising 632 early career registered nurses. Data were collected between November and December 2018 using an electronic survey with internationally validated measures including the Organizational Justice Scale, the Nurse Stress Index Scale, the Job Content Questionnaire, and the Sleep Problems Questionnaire. Latent profile analysis was used to identify groups with similar psychosocial work characteristic profiles. Multinomial and linear regression analyses were used to examine the association between latent work characteristics profiles and sleep quality.

**Results:**

Analysis yielded four profiles. The profiles were named based on the descriptions of classes as high strain/low justice, medium strain/high justice, medium strain/medium justice, and low strain/high justice. The low strain/high justice profile group (p = < 0.001) and the medium strain/high justice profile group (p = 0.002) had statistically significantly better sleep quality compared to the high strain/low justice profile group.

**Conclusions:**

High procedural and interactional justice may alleviate strain in early career registered nurses and protect them against sleep problems. Promoting organizational justice in early career stages seems an efficient way to enhance registered nurses’ well-being and sleep quality.

## Background

The quality of psychosocial work characteristics is critical for employees’ well-being in organizations [1]. Psychosocial work characteristics refer to factors involved with psychological processes linked to the social environment of work that may contribute to the onset of illness [2]. The current shortage of registered nurses exposes a need to promote psychosocial work characteristics that enable early career nurses to stay healthy throughout their careers [3]. Based on our definition, an early career registered nurse (hereinafter referred to as ECRNs) is a nurse who has been registered for up to and including five years [4]. An association has been found between psychosocial work characteristics and ECRNs’ health and well-being [5], including sleep problems [6]. Those ECRNs who have experienced a more challenging and stressful transition period to working life may have had more severe sleep problems during the transition [7]. Stressful psychosocial factors have been shown to be negatively associated with organizational commitment, productivity, and the quality and safety of patient care [8] and may contribute to turnover intention in early career [9].

Two of the widely tested epidemiological models defining psychosocial work characteristics are the job strain (or the job demand-control) model [10, 11] and the organizational justice model [12]. The job strain model includes two main components: job demand (the need to work quickly and hard) and job control (control over skill use, time allocation, and organizational decisions). Based on this model, employees who have concurrent low job control and high demands are not able to moderate their stress (caused by high demands) through time management or by learning new skills [10, 11]. Persistent exposure to stress at work also puts them at a higher risk to develop psychological distress and diseases. The job demand-control hypothesis has been confirmed among ECRNs [13] and other healthcare professionals [14]. Moreover, in a review examining demands related to the nursing work environment and health effects, Dall’Ora et al. (2020) identified several factors including a high workload, low control over the job, low decision latitude, and a lack of social support as predictors of poor health among the nursing workforce [15]. Similarly, other studies have demonstrated that, if prolonged, particularly high workloads and time pressures will result in health problems and stress [16] among ECRNs [5, 17]. A follow up-study also found that a high level of stress among ECRNs during their early career was significantly related to more frequent cognitive dysfunction and impaired sleep later in their careers [6].

Organizational justice refers to the fairness of the decisions an organization makes (distributive justice), the procedures it uses in making decisions (procedural justice), and the quality of the interpersonal interaction employees receive from their organization (interactional justice). Some researchers have further divided the interactional justice dimension into interpersonal and informational dimensions [18–20]. Research in organizational justice among registered nurses has focused on certain topics in previous years, including attitudes (such as job satisfaction), behaviors (such as job performance), and productivity [21, 22]. Furthermore, an increasing amount of evidence suggests that organizational justice and injustice are associated with a large variety of employee health outcomes [23, 24]. High organizational justice has been related to work ability [25], and a lower incidence of sleeping problems [26–28] and psychological distress [27, 29] among nurses. Meanwhile, low organizational justice has been found to be associated with poor self-rated health, health complaints [12, 30, 31], cardiovascular diseases, mortality, sickness absences, and a greater incidence of minor psychiatric disorders [12, 30, 32].

Organizational injustice and job strain have also been related to sleep problems. It is currently unknown how quickly a feeling of injustice starts to negatively affect sleep [33]. However, in a prospective longitudinal study, the effect of prolonged exposure to low organizational justice resulted in poor sleep quality when measured several years later [34]. Another longitudinal study showed that low procedural and interactional justice were associated with sleep problems in healthcare organization employees [35]. Plausible mechanisms linking injustice and job strain to sleep problems include prolonged stress and depression [34]. Lallukka et al. (2017) reported that changes in interactional justice may affect employees’ sleep quality. In their follow-up study conducted in the public sector including mostly healthcare professionals, they concluded that reduced interactional justice was linked to increased sleep problems, whereas increased interactional justice was associated with reduced sleep problems [36].

As the transition to working life has been identified as a challenging period [37] and sleep problems as a factor that impairs health among ECRNs [6, 7], further investigation on this topic would be important. Most previous studies on psychosocial work characteristics and ECRNs’ well-being have investigated psychosocial work characteristics using traditional correlation or regression methods that tend to neglect person-centered and latent analytic approaches [38]. It has been noted that person-centered and latent analytic approaches can offer new perspectives to explaining perceptions related to work characteristics [39] and health outcomes, such as sleep quality, among registered nurses [40]. Therefore, it would be interesting to investigate what kinds of latent patterns of work characteristics can be observed among ECRNs and which features strengthen or impair the sleep quality of ECRNs. There is still a need to extend the current knowledge in the field.

## Methods

### Aim

The aim of this study was to identify latent work characteristic profiles according to procedural, interactional and distributive justice, job demand, and job control, and to examine whether the profiles are associated with sleep quality. The research questions were:

1. Which work characteristic profiles can be identified among ECRNs?
2. Does the ECRN’s age, sex, work experience, work schedule, work unit and sector predict membership in a certain work characteristic profile?
3. Are work characteristic profiles associated with sleep quality among ECRNs?

### Sample

The present study is a cross-sectional survey conducted in Finland under one part of the larger Competent Workforce for the Future (COPE) research project. All participants were registered nurses who had graduated within two years before the present study (between September 2016 and June 2018). The total research sample (n = 6,797) was extracted from the Finnish Central Register of Valvira (National Supervisory Authority for Welfare and Health). The Union of Health and Social Care Professionals in Finland (Tehy) sent an invitation letter to participate in this study to 3,942 nurses whose email addresses it obtained from its register. The letter included a link to the electronic survey. Three email reminders were sent. Data collection took place between 1 November and 21 December 2018. In total, 712 nurses filled out the research survey. After removing missing data, the results of 632 nurses were analyzed, making the response rate 16 per cent. This can be considered acceptable [41].

### Measurements

*Organizational justice* was measured using an 8-item scale (e.g., *“The procedures used in my organization have been applied consistently”*) derived from the short version of the Organizational Justice Scale [42, 43] measuring procedural (3 items), interactional justice (3 items), and distributive justice (2 items). The items were rated on a five-point Likert scale ranging from 1 (totally disagree) to 5 (totally agree). The Cronbach’s alphas were 0.79 (procedural justice), 0.92 (interactional justice), and 0.92 (distributive justice) in this study.

*Job demand (workload)* was measured using a 3-item scale (e.g., “*How often constant rush and pressure due to uncompleted work has disturbed, worried or stressed you during the last two months”)* derived from the Nurse Stress Index [44] measuring excessive workloads and time pressures. The items were rated on a five-point Likert-scale ranging from 1 (hardly ever) to 5 (very often or continuously). The Cronbach’s alpha was 0.90 in this study.

*Job control* was measured using a 3-item scale (e.g., “*My job allows me to make a lot of decisions of my own”)* derived from the Job Content Questionnaire [45] measuring the freedom to make independent decisions. The items were rated on a five-point Likert scale ranging from 1 (totally disagree) to 5 (totally agree). One item was reverse coded. The Cronbach’s alpha was 0.64 in this study.

*Sleep quality* was measured using a 4-item scale (e.g., “*How often during the past weeks have you e.g. woken up feeling tired and worn out after the usual amount of sleep”*) derived from the Sleep Problems Questionnaire [46]. The items were rated on a six-point Likert scale ranging from 1 (not at all) to 6 (every night). The Cronbach’s alpha was 0.83 in this study.

### Data analysis

We used the Latent Profile Analysis (LPA), a person-centered approach that aims to identify subgroups of individuals who share a similar profile of scores on various indicators of interest [38]. To assess the profile indicator variables, we measured mean value variables of procedural justice, interactional justice, distributive justice, job demands, and job control. In LPA process, multiple fit values and content decision criteria should be applied when determining the final profile solution. We used the Akaike Information Criterion (AIC), Bayesian Information Criterion (BIC), and an entropy test for our model selection [47]. We utilized a stepwise approach to determine the number of latent profiles that best characterize the data and sample, starting with an LPA with two profiles and subsequently adding profiles [48]. In each step, we investigated the fit information criteria. Multinomial logistic regression was used in the analysis to examine the associations of age, sex, work experience, work schedule, work unit and work sector with profile class membership. Linear regression analysis was used to examine the association between profile class memberships and sleep quality [47]. The analyses were conducted using the R statistical software, version 4.2.1.

## Results

### The profiles of psychosocial work characteristics

According to the AIC, BIC, and entropy test values, the four-class and six-class models offered the best fit (Table 1). We selected the four-class model as the optimal choice based on the BIC and Entropy values and a relatively small difference in the AIC values between the four-class and six-class models [48].

**Table 1:** Model selection

Classes	AIC	BIC	Entropy
2	8441.06	8512.24	0.73
3	8268.14	8366.02	0.764
4	8184.03	8308.6	0.782
5	8185.43	8336.69	0.734
6	8130.67	8308.63	0.763

*Note*: AIC = Akaike Information Criterion, BIC = Bayesian Information Criterion, Entropy = Entropy test

**Fig. 1:**
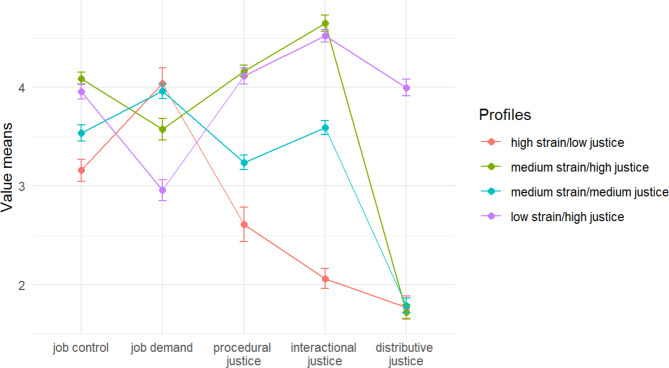
The class descriptions of psychosocial work characteristics profiles

The class descriptions are based on comparisons between psychosocial work characteristic indicators, including job demand, job control, procedural justice, interactional justice, and distributive justice. These indicators are presented as mean values and standard deviations (Fig. 1). The profiles were named based on the descriptions of classes as high strain/low justice (class 1), medium strain/high justice (class 2), medium strain/medium justice (class 3), and low strain/high justice (class 4). The high strain/low justice profile showed a low level of job control, a high level of job demand, and a low level of organizational justice in all three indicators. The medium strain/high justice profile was characterized by a high level of job control, a medium level of job demand, a medium level of procedural and interactional justice, and a low level of distributive justice. The medium strain/medium justice profile was characterized by an average level of job control and job demand, a medium level of procedural and interactional justice, and a low level of distributive justice. The low strain/high justice profile presented a high level of job control and a low level of job demand, and a high level of justice in all three organizational justice indicators.

### Membership in the work characteristic profile and sleep quality

The personal and work characteristics of the ECRNs are summarized in Table 2. Most participants in the total sample were female (90%) and their mean age was 31.01. Over half of the participants had worked in three shifts, in the hospital and in the public sector. Profile groups appeared as follows: the high strain/low justice profile (n = 85, 13.45%), the medium strain/high justice profile (n = 144, 22.78%), the medium strain/medium justice profile (n = 268, 42.41%), and the low strain/high justice profile (n = 135, 21.36%). Age, work experience, work schedule, work unit, and sector were not associated with certain work characteristic profiles (Table 3). Male gender slightly predicted belonging to the low strain/high justice profile. The low strain/high justice profile group (p = < 0.001) and the medium strain/high justice profile group (p = 0.002) had the best sleep quality with high statistical significance compared to the high strain/low justice profile group. The sleep quality of those with a medium strain/medium justice profile group (p = 0.287) was not statistically significantly better when compared to the high strain/low justice profile (Table 4).

**Table 2:** ECRNs’ personal and work characteristic variables

	Entire sample	Class 1: high strain/low justice	Class 2: medium strain/high justice	Class 3: medium strain/medium justice	Class 4: low strain/high justice
n (%)	632	85 (13.45)	144 (22.78)	268 (42.41)	135 (21.36)
Sex					
Male	63 (9.97)	6 (7.06)	13 (9.03)	20 (7.46)	24 (17.78)
Female	569 (90.03)	79 (92.94)	131 (90.97)	248 (92.54)	111 (82.22)
Age					
20–29	389 (61.55)	52 (61.18)	87 (60.42)	165 (61.57)	85 (62.96)
30–39	137 (21.68)	23 (27.06)	28 (19.44)	58 (21.64)	28 (20.74)
40–49	68 (10.76)	8 (9.41)	16 (11.11)	31 (11.57)	13 (9.63)
50–	38 (6.01)	2 (2.35)	13 (9.03)	14 (5.22)	9 (6.67)
Work schedule					
One-shift (daytime)	142 (22.47)	19 (22.35)	32 (22.22)	59 (22.01)	32 (23.70)
Two-shift (morning and evening)	99 (15.66)	10 (11.76)	25 (17.36)	40 (14.93)	24 (17.78)
Three-shift (morning, evening, and night)	352 (55.70)	48 (56.47)	80 (55.56)	158 (58.96)	66 (48.89)
Other	39 (6.17)	8 (9.41)	7 (4.86)	11 (4.10)	13 (9.63)
Work experience					
1–5 months	137 (21.68)	17 (20)	35 (24.31)	48 (17.91)	37 (27.41)
6–11 months	229 (36.23)	27 (31.76)	53 (36.81)	105 (39.18)	44 (32.59)
1–2 years	221 (34.97)	31 (36.47)	43 (29.86)	100 (37.31)	47 (34.81)
3 years and over	45 (7.12)	10 (11.76)	13 (9.03)	15 (5.6)	7 (5.19)
Current work unit					
Elderly care	43 (6.80)	7 (8.24)	10 (6.94)	15 (5.60)	11 (8.15)
Hospital	370 (58.54)	45 (52.94)	88 (61.11)	168 (62.69)	69 (51.11)
Outpatient	197 (31.17)	30 (35.29)	43 (29.86)	78 (29.10)	46 (34.07)
Other	22 (3.48)	3 (3.53)	3 (2.08)	7 (2.61)	9 (6.67)
Sector					
Public	534 (84.49)	70 (82.35)	119 (82.64)	235 (87.69)	110 (81.48)
Private	82 (12.97)	14 (16.47)	19 (13.19)	29 (10.82)	20 (14.81)
Third	16 (2.53)	1 (1.18)	6 (4.17)	4 (1.49)	5 (3.70)

## Discussion

The purpose of this study was to identify latent work characteristic profiles according to procedural, interactional, and distributive justice, job demand, and job control, and examine whether the profiles were associated with sleep quality among ECRNs. LPA provided a novel approach studying psychosocial work environment and ECRNs sleep quality. We identified four work characteristic profiles. These work characteristic profiles were guided by the demand-control model [11] and organizational justice model [12]. Previous studies recommended considering not only statistical fit values but also theoretically coherent and content-related aspects when selecting profile groups [49]. Based on these considerations, this study provides a meaningful perspective on ECRNs’ work characteristic profiles.

**Table 3:** The associations between personal and work characteristics to class profiles

	Class 2: medium strain/high justice vs. Class 1: high strain/low justice			Class 3: medium strain/medium justice vs. Class 1: high strain/low justice			Class 4: low strain/high justice vs. Class 1: high strain/low justice		
Predictors	OR	CI	p	OR	CI	p	OR	CI	p
(Intercept)	0.98	0.07–14.28	0.988	1.99	0.16–24.13	0.587	7.31	0.56–94.70	0.128
Sex (Men=ref.)	0.73	0.26–2.06	0.556	0.89	0.34–2.36	0.823	0.32	0.12–0.85	0.022
Age	1.02	0.99–1.06	0.203	1.01	0.98–1.04	0.61	1.01	0.98–1.05	0.399
Work experience									
1-5 months	1.54	0.72–3.28	0.264	0.89	0.45–1.79	0.75	1.54	0.73–3.27	0.256
6-11 months	1.4	0.72–2.71	0.321	1.18	0.65–2.12	0.59	1.07	0.55–2.10	0.839
1-2 years	ref.	ref.	ref.	ref.	ref.	ref.	ref.	ref.	ref.
3 years and over	0.84	0.30–2.37	0.745	0.51	0.19–1.35	0.176	0.37	0.12–1.19	0.096
Work schedule									
One-shift (daytime)	ref.	ref.	ref.	ref.	ref.	ref.	ref.	ref.	ref.
Two-shift (morning and evening)	1.27	0.48–3.38	0.627	1.18	0.48–2.91	0.722	1.35	0.51–3.58	0.545
Three-shift (morning, evening, and night)	0.64	0.28–1.47	0.295	0.66	0.31–1.40	0.278	0.71	0.31–1.61	0.407
Other	0.35	0.09–1.31	0.12	0.34	0.10–1.11	0.075	0.82	0.24–2.84	0.758
Current work unit									
Elderly care	ref.	ref.	ref.	ref.	ref.	ref.	ref.	ref.	ref.
Hospital	2.26	0.66–7.51	0.194	2.13	0.70–6.47	0.18	1.2	0.37–3.92	0.764
Outpatient	1.1	0.35–3.49	0.873	1.12	0.39–3.24	0.829	0.97	0.31–2.99	0.951
Other	0.63	0.09–4.67	0.654	1.16	0.21–6.41	0.861	1.63	0.29–9.08	0.577
Sector									
Public	1.05	0.45–2.45	0.908	1.32	0.61–2.88	0.479	1.16	0.50–2.68	0.735
Private	ref.	ref.	ref.	ref.	ref.	ref.	ref.	ref.	ref.
Third	6.45	0.65–64.47	0.112	2.49	0.24–26.00	0.444	3.52	0.34–36.25	0.29

The members of the high strain/low justice profile with high workloads and low job control also experienced low organizational justice. Factors related to a negative work environment, such as a heavy workload, can trigger negative effects on employees. The duration of workloads has been thought to be partially linked to how individuals perceive and are able to regulate their workloads [16]. In the long term, a high workload can result in stress. Stressed employees feel less equipped to apply adaptive coping strategies, such as prioritizing tasks and rearranging duties. Experiences of stress may reduce an employee’s sense of capability or actual capacity to cope at work [50]. Instead, mixed results have been presented regarding the effects of age on perceived stress [51]. Some ECRNs may nevertheless perceive their environment as more strenuous and demanding than others. For example, ECRNs may face multiple situations of uncertainty at the beginning of their careers [52]. In times of uncertainty, employees pay special attention to the fairness of any judgments by their management. Fairness matters to employees because it helps them cope with uncertainty [53, 54].

**Table 4:** The associations of class profiles to sleep

Predictors	Estimates	95% CI	p
(Intercept)	2.92	2.69–3.15	< 0.001
Class 1: high strain/low justice	Ref.	Ref.	Ref.
Class 2: medium strain/high justice	-0.45	-0.75 – -0.16	0.002
Class 3: medium strain/medium justice	-0.14	-0.41–0.12	0.287
Class 4: low strain/high justice	-0.68	-0.97 – -0.38	< 0.001

*Note*: CI = Confidence intervals

The members of low strain/high justice and medium strain/high justice profiles presented lower workloads and a higher level of job control than the other groups. Job control is considered to act as a protective factor to alleviate the burdensome effect of work demands [11]. For example, Chiu et al. (2013) pointed out in their study that increased job control compensated for the high demands of work in registered nurses [55]. Furthermore, high organizational justice has been shown to strengthen the buffering role of job control on high job demands in nursing [56] which is in line with our findings.

ECRNs’ experiences of low distributive justice were highly similar in the high strain/low justice profile, in the medium strain/high justice profile, and the medium strain/medium justice profile groups. This finding may be partly explained by the perception that employees who experience injustice because of stress at work also consider rewards as unfair or vice versa [19]. Pay is often seen as a strong indicator of justice [57]. In these profile groups, ECRNs may generally feel that their pay level is low in relation to demands which has been found to be a source of job dissatisfaction among nurses [58]. In the low strain/high justice profile group, distributive justice was higher than in the other groups. There is evidence suggesting that high distributive justice was associated with a lower level of stress symptoms in registered nurses [59]. Male gender somewhat predicted membership in the low strain/high justice profile. There is evidence suggesting differences between perceptions of justice among female and male employees [60]. However, we are not able to draw strong conclusions in this context due to the relatively small number of male participants.

Perceived sleep quality was substantially better in the low strain/high justice and medium strain/high justice profiles. Similar results from healthcare organizations have been reported in previous cross-sectional studies conducted among registered nurses [26, 27] and in longitudinal studies carried out among physicians [14]. Those ECRNs with a high strain/low justice profile perceived their sleep quality as poor. This finding is in line with a systematic review that reported that low organizational justice manifested as elevated sleeping problems [24]. Meanwhile, the negative health effects (such as sleep quality) of low organizational justice are more intense in unpredictable and uncertain situations [53, 61]. It is worth noting that changing management to involve fair interaction has the potential for improving employees’ sleep quality [36].

### Limitations

Although this study has several strengths, there are also limitations. Our cross-sectional study does not lend itself to making any causal interpretations of psychosocial work characteristics and their relation to sleep quality or the direction of the associations. Based on the response rate of 16%, this study sample is not necessarily representative of the population as a whole. However, this study provides an overview of ECRNs’ profiles working in different shifts, work units, and sectors. The Cronbach’s alpha of job control was 0.64 in this study. This should be taken into account when interpreting the results. The present study includes a limited range of psychosocial work characteristics or other factors that may increase or decrease ECRNs’ stress and be further associated with sleep quality. The data used in this study were acquired prior to Covid-19 pandemic. We cannot exclude that the findings could differ in the post-Covid-19 era. However, it is plausible to assume that policies supporting ECRNs health and well-being, including organizational justice, are still key elements of leadership.

## Conclusions

Based on the findings from the profile groups, ECRNs who experienced a pattern of high organizational justice, high job control and low job demands reported better sleep quality. Meanwhile, ECRNs who perceived low organizational justice and high job demands and low job control tended to rate their sleep quality as poor. Organizational justice may support ECRNs and further strengthen their well-being, including sleep quality. Further research is needed on whether organizational justice, job demand, job control, and other health- or work-related outcomes, teamwork, and individual or structural characteristics are patterned and associated with these profiles.

Organizational justice seems to provide one of the efficient ways to enhance ECRNs’ health and well-being. Nurse managers and leaders play a crucial role in promoting organizational justice in the early career stages of RNs. Furthermore, nurse managers and leaders may mitigate ECRNs high job demands and low job control by work arrangements such as work scheduling, well-planned and individualized orientation, feedback discussions, and adequate daily support. Special importance should be paid to identify and support those ECRNs at risk for strain. Taking this into account by training managers and in everyday management may benefit ECRNs.

## Data Availability

The datasets used and/or analysed during the current study are available from the authors on reasonable request.

## Abbreviations

AIC: Akaike Information Criterion
BIC: Bayesian Information Criterion
CI: Confidence Interval
ECRN: Early career registered nurse
LPA: Latent Profile Analysis
RN: Registered nurse
